# Orthotopic transplantation of the bioengineered lung using a mouse-scale perfusion-based bioreactor and human primary endothelial cells

**DOI:** 10.1038/s41598-024-57084-0

**Published:** 2024-04-04

**Authors:** Fumiko Tomiyama, Takaya Suzuki, Tatsuaki Watanabe, Jun Miyanaga, Anna Suzuki, Takayasu Ito, Sho Murai, Yuyo Suzuki, Hiromichi Niikawa, Hisashi Oishi, Hirotsugu Notsuda, Yui Watanabe, Takashi Hirama, Ken Onodera, Takeo Togo, Masafumi Noda, Thomas K. Waddell, Golnaz Karoubi, Yoshinori Okada

**Affiliations:** 1 Department of Thoracic Surgery, Institute of Development, Aging and Cancer, Tohoku University, 4-1 Seiryo-machi, Aoba-ku, Sendai, Miyagi 980-8575 Japan; 2 Institute of Fluid Science, Tohoku University, 2-1-1 Katahira, Aoba-ku, Sendai, Miyagi 980-8577 Japan; 3 Latner Thoracic Research Laboratories, Toronto General Hospital Research Institute, University Health Network, 101 College Street, Toronto, ON M5G1L7 Canada; 4 Institute of Medical Science, Faculty of Medicine, University of Toronto, 1 King’s College Circle, Toronto, ON M5S1A8 Canada; 5 Department of Laboratory Medicine and Pathobiology, Faculty of Medicine, University of Toronto, 1 King’s College Circle, Toronto, M5S1A8 Canada

**Keywords:** Lung bioengineering, Vascular engineering, Decellularization, Perfusion-based Bioreactor, Transplantation, Box-counting analysis, Tissue engineering, Applied mathematics

## Abstract

Whole lung engineering and the transplantation of its products is an ambitious goal and ultimately a viable solution for alleviating the donor-shortage crisis for lung transplants. There are several limitations currently impeding progress in the field with a major obstacle being efficient revascularization of decellularized scaffolds, which requires an extremely large number of cells when using larger pre-clinical animal models. Here, we developed a simple but effective experimental pulmonary bioengineering platform by utilizing the lung as a scaffold. Revascularization of pulmonary vasculature using human umbilical cord vein endothelial cells was feasible using a novel in-house developed perfusion-based bioreactor. The endothelial lumens formed in the peripheral alveolar area were confirmed using a transmission electron microscope. The quality of engineered lung vasculature was evaluated using box-counting analysis of histological images. The engineered mouse lungs were successfully transplanted into the orthotopic thoracic cavity. The engineered vasculature in the lung scaffold showed blood perfusion after transplantation without significant hemorrhage. The mouse-based lung bioengineering system can be utilized as an efficient ex-vivo screening platform for lung tissue engineering.

## Introduction

Lung transplantation has been an established and decisive treatment for end-stage lung diseases caused by a range of respiratory illnesses, such as idiopathic pulmonary fibrosis or chronic obstructive pulmonary disease. The donor organ shortage, however, remains a major obstacle to this life-saving treatment^1^. Bioengineering is the potential tool to create a functional donor organ by utilizing immune-compatible cells and a scaffold, which could be a solution to potentially overcome not only the donor shortage in transplantation, but reduce transplant-related complications including graft rejection, size mismatch, or variable graft function^2^. Organ decellularization and recellularization is an emerging approach to create bioengineered lungs with the goal to recapitulate functional organs in vitro for clinical transplantation^3,4^.

Although previous pioneering investigations have demonstrated the potential utility of bioengineered lungs for clinical transplantation^3,4^, the lack of relevant and low-cost experimental platforms has been hindering stepwise research and development, which would facilitate the translation and scale-up to large, human-scale organ bioengineering^5^. Rat or swine lungs have previously used for perfusion bioreactor setups where the trachea, pulmonary artery and pulmonary vein were cannulated to perform mechanical ventilation and media perfusion using a syringe pump and a pulsatile pump^3–9^. These systems, however, require quite a large number of cells. Using smaller mouse-based systems can reduce the size of each experiment, both in terms of cell number and bioreactor system. A smaller-scale system would accelerate the optimization process of lung tissue engineering. Previous studies have utilized decellularized mouse lungs for cell repopulation; however, the culture conditions were either static using decellularized lung slices^10^ or not physiologically relevant where mouse lungs were incubated in a rotating syringe^11^. Recent literature has reported that a mouse-scale dynamic culture platform could enhance the repopulation of epithelial cells in decellularized mouse lung scaffolds, which led us to extend the platform to vascular engineering with proper vascular access^12,13^. Herein, we expand on this platform to optimize and evaluate the endothelialization of acellular mouse lung scaffolds.

Engineering intact vasculature in highly vascularized organs such as the lung remains a major challenge^14^. This requires numerous endothelial cells to cover the extremely large blood-gas interface in a meticulously coordinated manner to establish the immediate transport of nutrients and gas after transplantation. These functionalities could not be tested in simple two-dimensional culture conditions or in static three-dimensional models, which do not necessarily recapitulate the physiological environment of the complex organ. The aim of this study is to develop a three-dimensional *ex-vivo* organ culture system replicating the physiological environment of the lung in a cost-effective way. To this end, we employed the mouse, one of the most common experimental animals. The bio-engineered lungs created in this mouse-scale system can be used to test not only cellular phenotype using gene expression analysis or histological analysis but also vascular functionality by direct transplantation of the bioengineered products.

## Results

**Figure 1:**
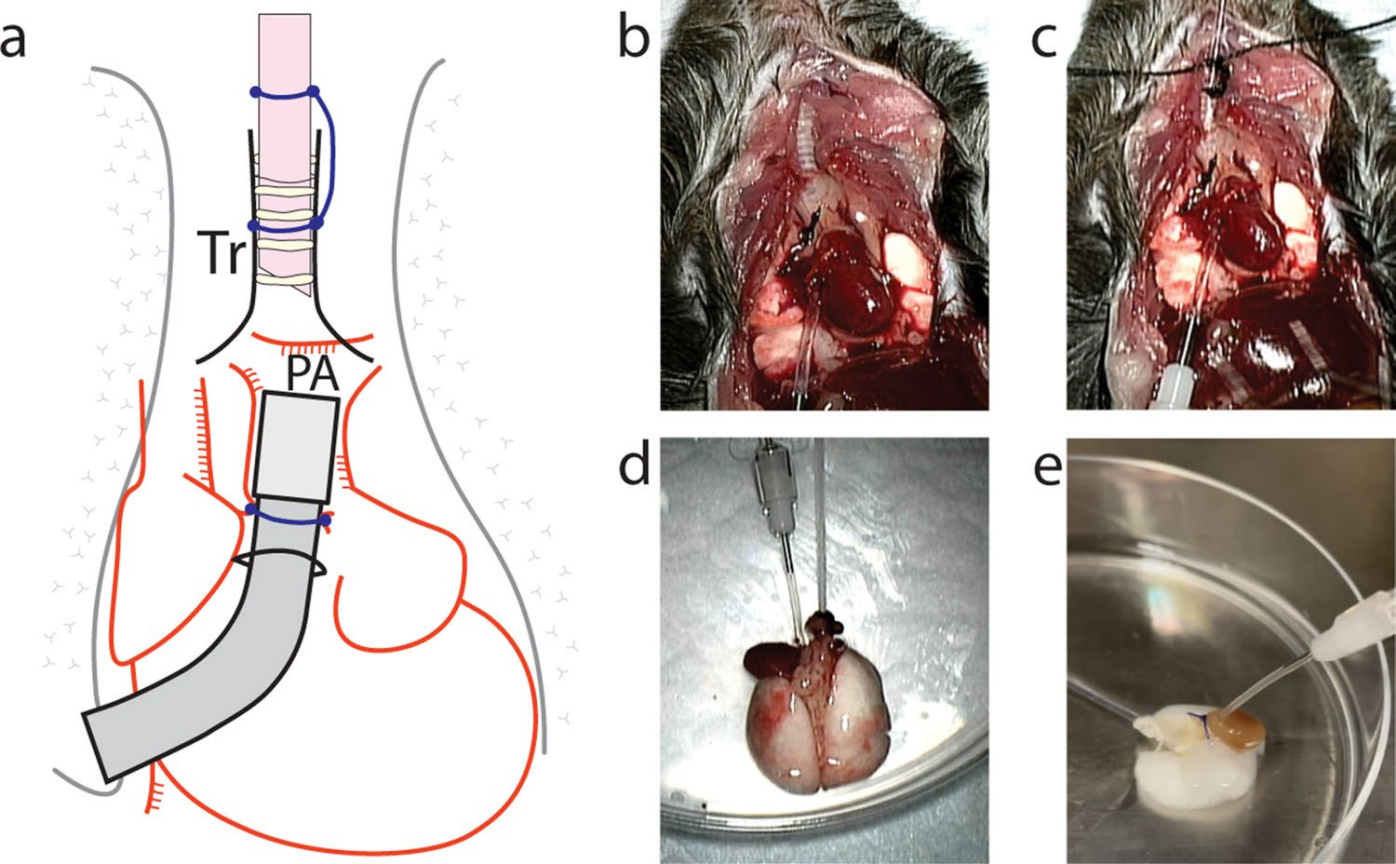
Decellularization of the mouse heart–lung block. Cannulation layout for the mouse heart–lung block (**a**). Representative images during the microscopic surgery (**b** and **c**). The pulmonary artery (PA) catheter was inserted via a window of the right ventricle (**b**). The tracheal catheter was inserted and secured just below the larynx (**c**). The cannulated heart–lung block was removed from the thoracic cavity (**d**). Sequential detergent administration was performed from the PA cannula and tracheal cannula.

**Figure 2:**
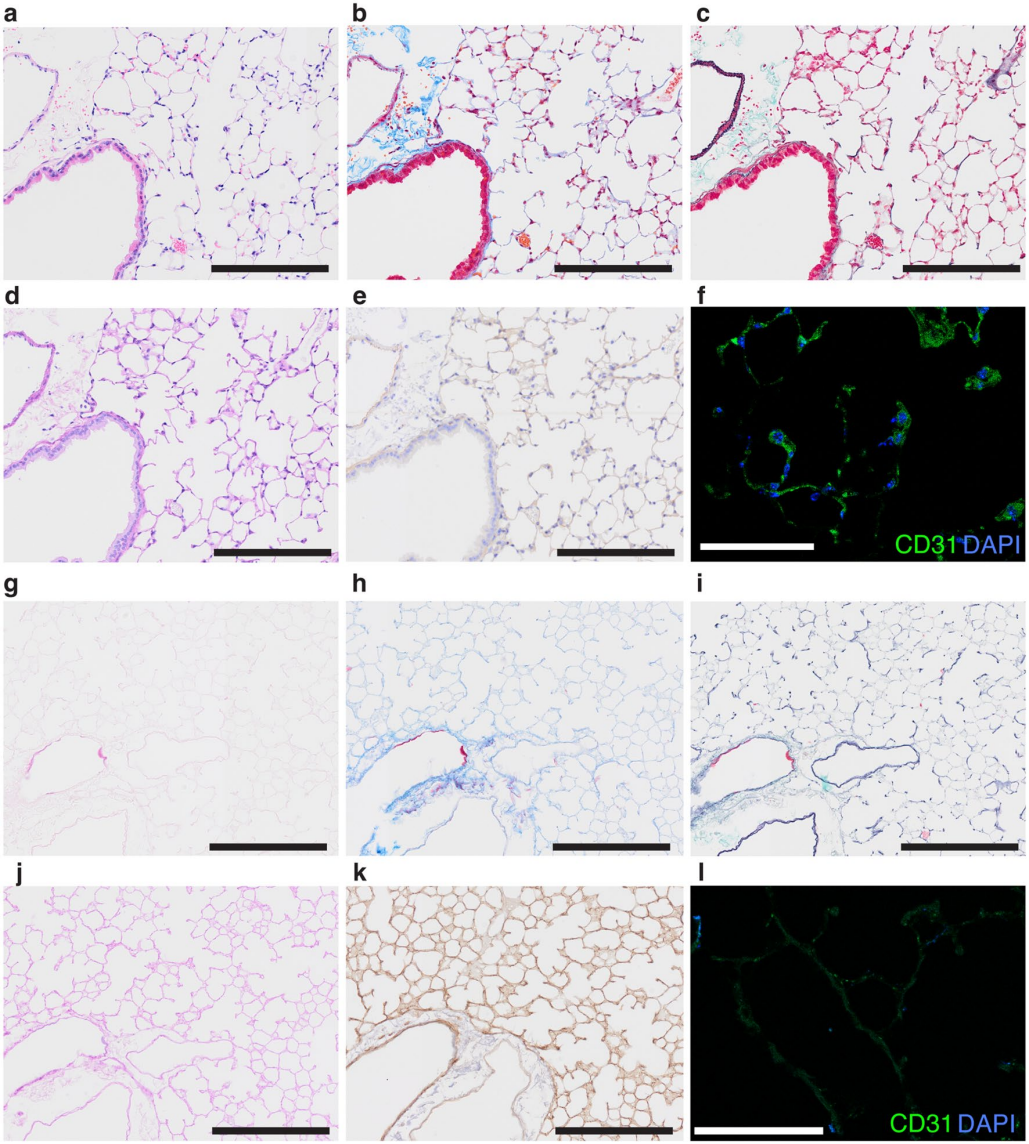
Representative histological images of the native mouse lung and the decellularized mouse lung. Native lungs (**a**–**f**). Decellularized lungs (**g**–**l**). Hematoxylin–eosin staining (**a** and **g**). Masson-trichrome staining (**b** and **h**). Elastica-Masson staining (**c** and **i**). Periodic-acid-Schiff staining (**d** and **j**). Immunohistochemistry of laminin (**e** and **k**). Immunofluorescence of CD31 (**f** and **l**). Bars; 200 µm (**a**–**e**, **g**–**k**), bars; 100 µm (**f** and **l**).

The cannulation procedure to secure access routes to the airway and pulmonary vasculature was successfully performed under stereo microscope surgery (Fig. 1a–c). The detailed procedure is described in Supplementary Fig. 1. Subsequent decellularization of the mouse heart–lung block (Fig. 1d) was completed through injection routes into both the airway and the pulmonary artery (Fig. 1e). The decellularized lung scaffold maintained a similar extracellular matrix (ECM) structure compared to the native lungs, while the cell components were removed (Fig. 2). ECM structures such as collagen fibers (Fig. 2b,c,h,i) and basement membrane (Fig. 2c,i) were maintained after the decellularization procedure. Water-soluble polysaccharide-rich components, which are stained as magenta in periodic-acid Schiff (PAS) staining, were removed during the decellularization process, as shown in Fig. 2d,j. Laminin expression remained intact with no morphological differences when compared to the native tissue (Fig. 2e,k). CD31-positive endothelial cells that were observed in the native lungs (Fig. 2f) were removed, and there was no detection of CD31 in the scaffold following the decellularization process (Fig. 2l).

**Figure 3:**
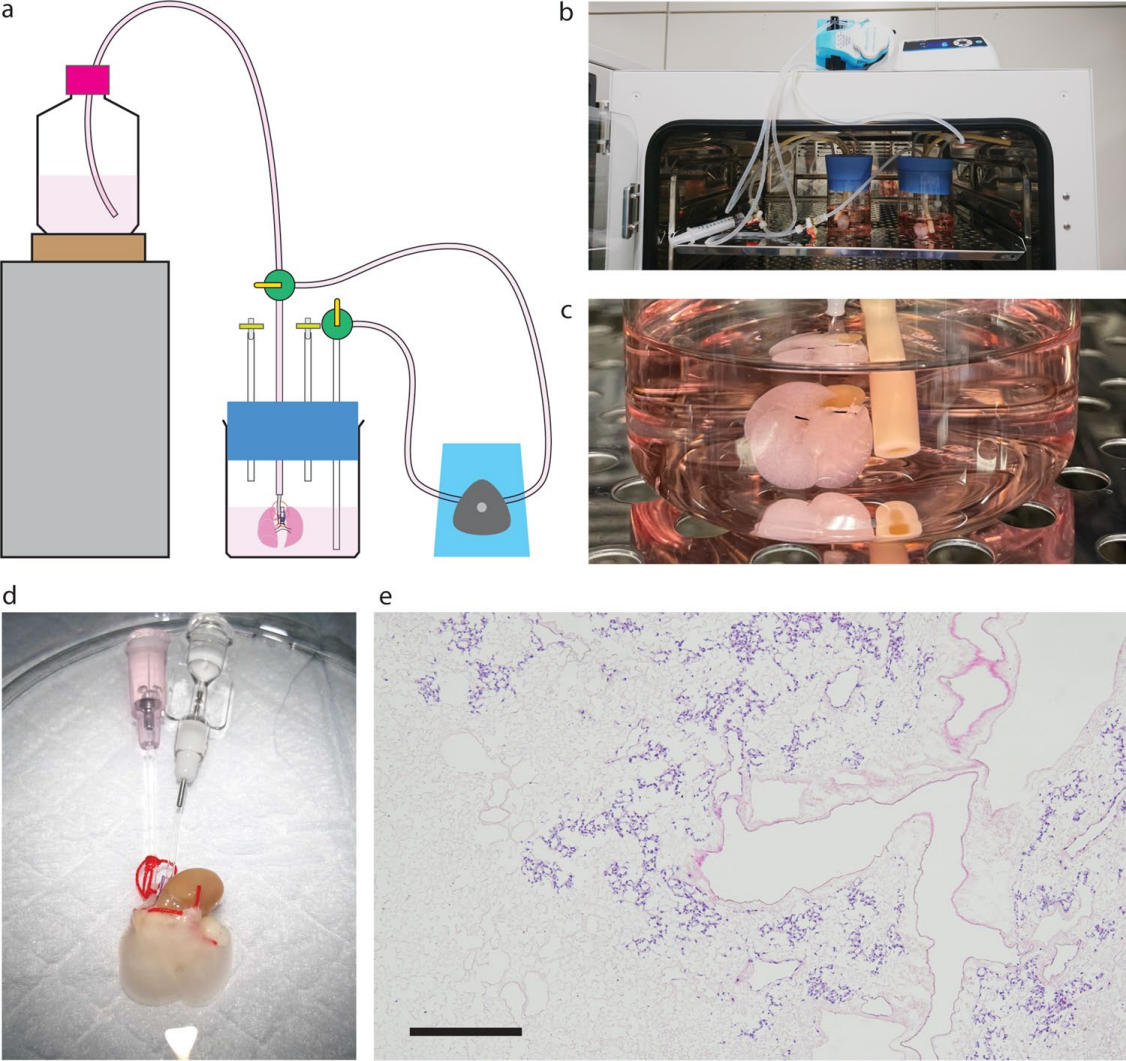
Perfusion-based bioreactor culture of decellularized mouse lungs. Schematic illustration of in-house developed perfusion-based bioreactor (**a**). Decellularized mouse lungs were connected to the media circuit and the culture media was perfused using a pulsatile pump at a speed of 2 mL/min (**b**). Decellularized mouse lungs were incubated for 2–3 days in a glass chamber (**c**). After the perfusion organ culture, the recellularized mouse heart–lung blocks were harvested (**d**). A representative image of recellularized mouse lungs using 10 million HUVECs (**e**).

The cannulated and decellularized mouse heart–lung blocks were then connected to the in-house developed perfusion-based bioreactor, which was assembled using commercially available materials (Fig. 3a). 1 × 10^7^ human umbilical cord vein endothelial cells (HUVECs) were injected into the decellularized scaffold through the catheter inserted into the main pulmonary artery in a gravity-driven manner. Perfusion culture was performed for three days (Fig. 3b,c). No tissue damage was observed after the perfusion culture in the bioreactor chamber (Fig. 3d). HUVECs appeared to attach preferentially to the proximal location of the decellularized scaffold (Fig. 3e).

**Figure 4:**
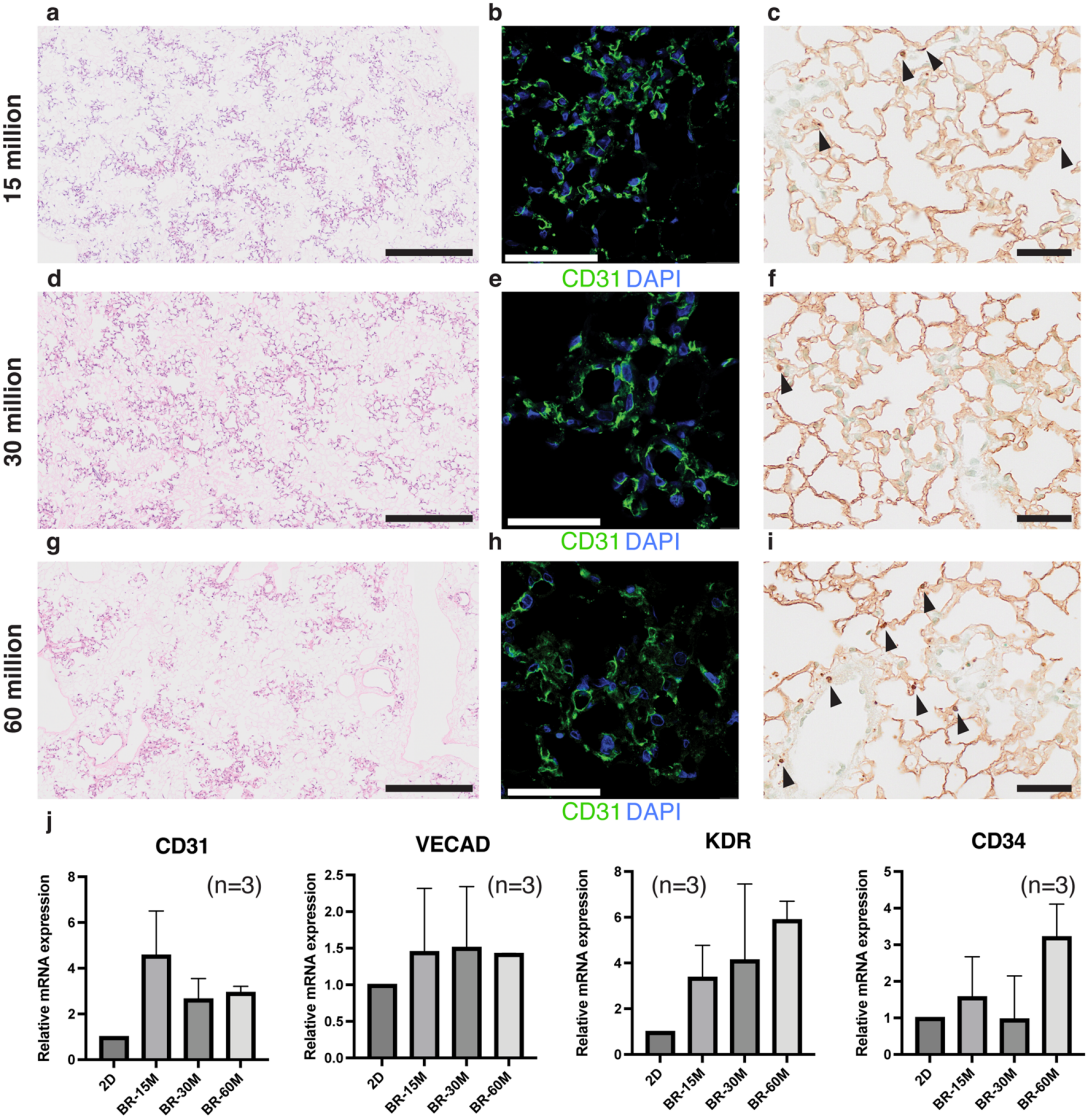
Comparison of revascularized lung scaffold using different numbers of HUVECs. 15 million (**a**–**c**), 30 million (**d**–**f**), or 60 million (**g**–**i**) of HUVECs were injected, and perfusion culture of the HLB was performed for 2 days. Representative images of hematoxylin–eosin staining (**a**, **d**, and **g**), immunofluorescence of CD31 (**b**, **e**, and **h**), and TUNEL staining (**c**, **f**, and **i**). Quantitative PCR was performed to quantify the gene expression difference of HUVECs before and after perfusion-bioreactor culture (**j**), from three biological replicate experiments. BR; Bioreactor. Arrowheads; TUNEL positive cells. Bars; 200 µm (**a**, **d**, and **g**), 50 µm (**b**, **c**, **e**, **f**, **h**, and **i**).

To optimize the number of cells to cover the pulmonary vasculature by HUVECs in the perfusion-based bioreactor culture, we injected different numbers of cells into the decellularized lung scaffold. Specifically, we compared 15 million, 30 million, and 60 million as indicated in Fig. 4. Numbers were selected based on previous literature estimated numbers of approximately 60 million lung endothelial cells in a mouse^15^. The number of cells that were not trapped in the lung scaffold at the initial injection were 1.95(± 0.28) × 10^6^, 3.91(± 0.31) × 10^6^, and 1.20(± 0.76) × 10^6^ when 1.5 × 10^7^, 3.0 × 10^7^, and 6.0 × 10^7^ cells were injected respectively. Hematoxylin–eosin (HE) staining, showed that cells were evenly distributed in the mouse decellularized lungs across the proximal pulmonary arteries to the peripheral capillary. Interestingly, injecting 60 million HUVECs did not seem to cover decellularized lungs any better than 30 million HUVECs (Fig. 4a,d,g). CD31 signals were similar across different conditions (Fig. 4b,e,h). The number of TUNEL-positive cells seemed highest in 60 million HUVECs injected (Fig. 4i), suggesting the increasing number of apoptotic cells in this group of scaffolds. To test if the difference in culture conditions between 2 and 3D perfusion culture or the difference in the number of cells injected would influence the endothelial gene expression, we performed quantitative PCR using the samples collected after a two-day culture period in a perfusion bioreactor with 2D-cultured cells used as a control. We found that the expression levels of endothelial cell-related genes such as CD31, VECAD, KDR, and CD34 tended to be higher when cultured in the perfusion-based bioreactor than when cultured in two-dimensional flat culture flasks (Fig. 4j). However, the differences were not statistically significant.

**Figure 5:**
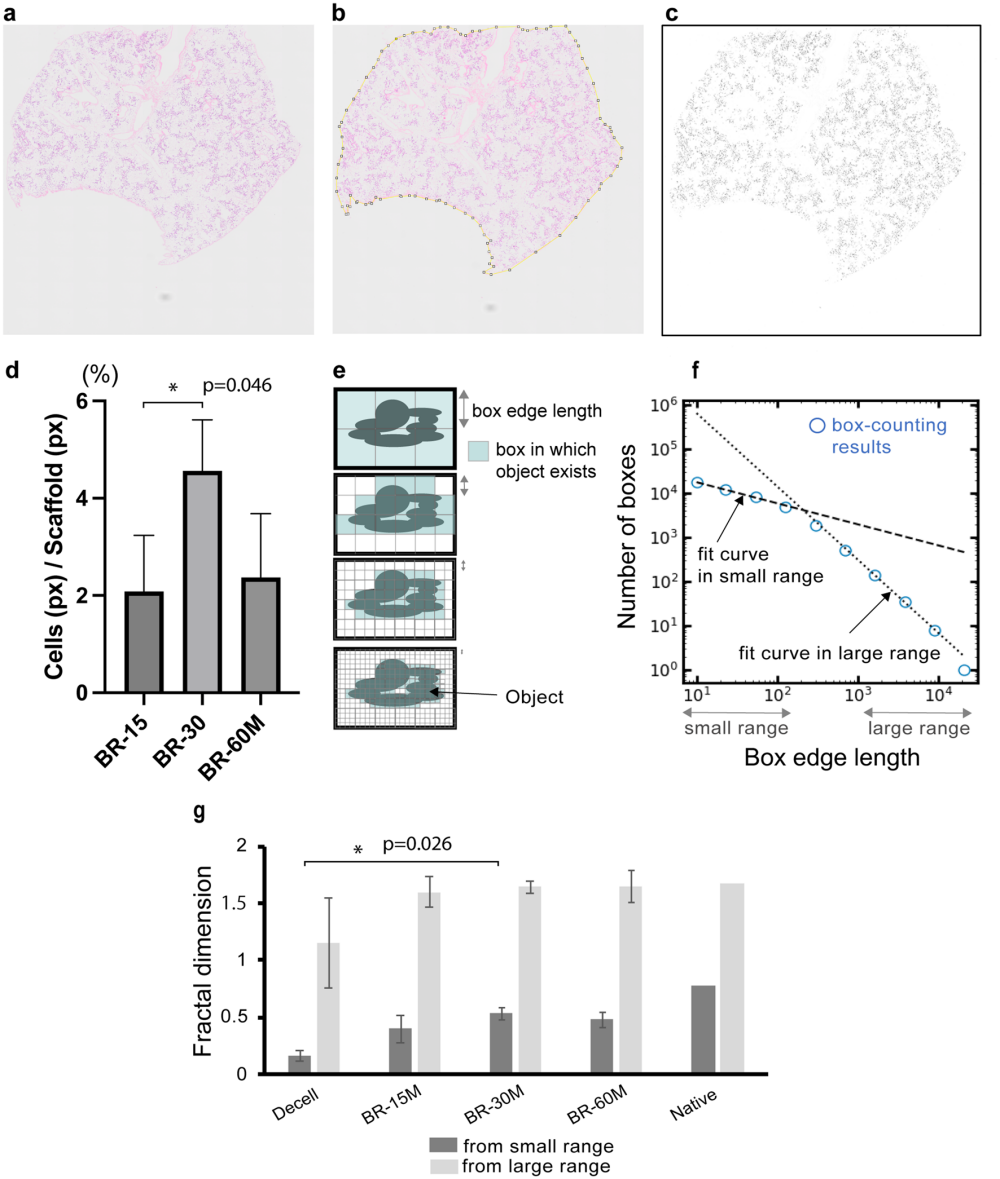
Fractal analysis of the recellularized lungs. A representative HE image of a recellularized lung (**a**). The scaffold area was selected, and the pixel numbers were counted (**b**). The HE images were converted into grayscale and binarized (**c**). The percentages of cell area in scaffold area are indicated in d. The schematic procedure of the box-counting methods (**e**). Log–log plotting of the box-counting obtained from the lung scaffold recellularized with 30 million HUVECs (**f**). Summary of fractal dimensions calculated from different samples (**g**).

Quantitative image analysis was performed to evaluate the number and the distribution of HUVECs after perfusion-based bioreactor culture. First, the vascular cell coverage of the decellularized scaffold was compared among the lung scaffolds that were revascularized using different numbers of cells. The mean percentage of cell area in the scaffold area was highest when 3.0 × 10^7^ HUVECs were injected and cultured. Then, to compare the structural complexity of the revascularized scaffolds to the native lungs or acellular scaffold, we employed a box-counting method for characterizing the cell distribution (Fig. 5e)^16^. The box-counting method is widely accepted and acknowledged to quantify self-similarity and fractal features of complex objects such as coastlines. Figure 5f shows an example of box counts for each box size in a log–log plot generated for the data collected in scaffolds repopulated with 30 million HUVECs. All analyzed images and results are shown in Supplementary Fig. 2. If the structure is self-similar and exhibits fractality, the result of box-counting shows one linear approximation and one dimension can be obtained. On the other hand, our results show two linear approximations representing two different box-counting dimensions in the same image. One was derived from counting the smaller boxes (microstructure), and the other was derived from counting the larger boxes (macrostructure). This trend is consistent across different culture conditions and a native lung (Supplementary Fig. 2). The box-counting dimensions of macrostructure represent the overall image spread and depends on the shape of the samples. On the other hand, the box-counting dimensions of microstructure represents the degree to which small structures (in this case cells) are agglomerated. The box-counting dimensions of microstructure ranged from 0.19 (Decell) to 0.78 (Native) (Fig. 5g). Of note, the box-counting dimensions for the microstructure of scaffolds repopulated with 30 million HUVECs were statistically different (Fig. 5g). Based on these image analyses, we concluded that 30 million HUVECs provided the best coverage and the most similar structural complexity to the native lung.

**Figure 6:**
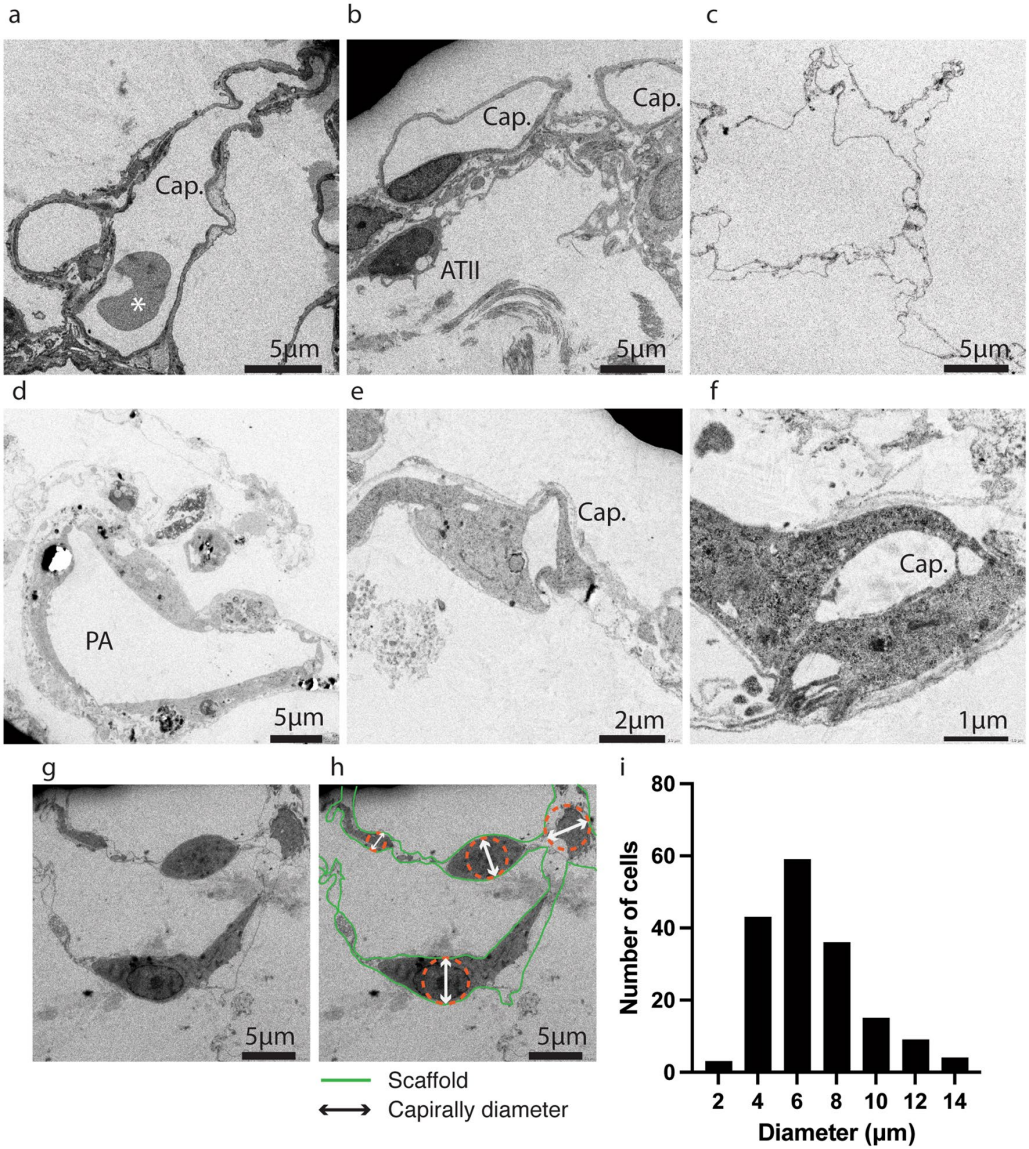
Transmission electron microscope of engineered mouse lungs. Representative high-magnification images of native mouse lungs (**a** and **b**), decellularized mouse lungs (**c**), and recellularized mouse lungs using 30 million HUVECs and perfusion-bioreactor (**d**–**h**). Panel h explains how the caliber of the vascular tube was measured. The number of cells that were attached to the vascular tube with a specific diameter (bin width = 2) is shown as a histogram (**i**). *Red blood cell, Cap.: Capillary endothelial cells, ATII: Alveolar Type II cells, PA: Pulmonary artery.

We then investigated the ultra-microstructure of the recellularized lungs and tested the functionality of the engineered pulmonary vasculature. Transmission electron microscope (TEM) imaging showed that injected HUVECs formed patent capillary lumens inside the microcapillary vasculature (Fig. 6). The diameters of revascularized capillary lumens ranged from approximately 2 to 10 µm (Fig. 6d–f), some of them were not sufficiently large enough to allow for the passage of red blood cells compared to the native lung capillaries (Fig. 6a). We also estimated the caliber of capillary vessels to which an individual HUVEC was attached by measuring the diameter of the inscribing circle within the blood vessel wall (Fig. 6g,h, and Supplementary Fig. 3). The caliber was measured in 169 positions. HUVECs were attached to the various capillary vessels raging from 2 µm to 15 µm, and the majority of the HUVECs were located in capillary vessels less than 10 µm in diameter (Fig. 6i).

**Figure 7:**
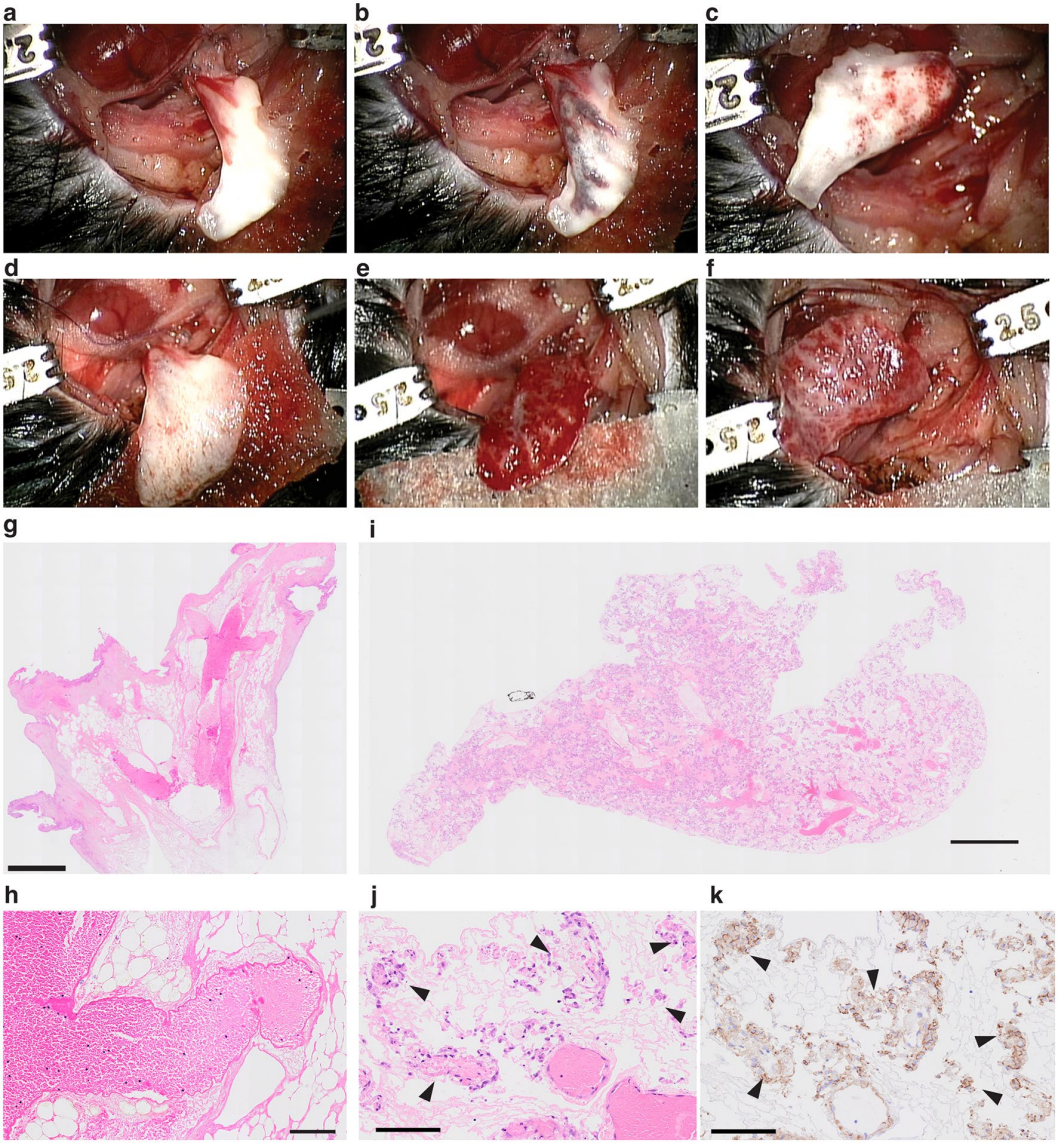
Orthotopic transplantation of revascularized mouse lung. Representative intraoperative images of left lung transplantation of decellularized lungs (**a**–**c**) and recellularized mouse lungs using 30 million HUVECs and perfusion-bioreactor (**d**–**f**). Each panel is; before de-cramping the pulmonary artery (**a** and **d**), the ventral side of the transplanted left lung 1 min after reperfusion, and the dorsal side of the transplanted left lung 1 min after reperfusion. Representative histological images of transplanted lungs (**g**–**l**). Hematoxylin–eosin staining (**g**, **h**, **i**, and **j**). Immunohistochemistry of CD31 (**k**). Bars; 500 µm (**g**), 1000 µm (**i**), 100 µm (**h**, **j** and **k**). Arrowheads; Red blood cells.

To confirm the endothelial cell integrity of the revascularized lung scaffold, we transplanted the left lung of the HUVEC-recellularized scaffolds after a two-day perfusion-bioreactor culture (decellularized left lungs were also transplanted as an acellular control (Fig. 7). When the decellularized lung was transplanted, the blood immediately filled the large proximal vessels of the transplanted decellularized lung (Fig. 7b) and did not circulate to the peripheral regions of the lung (Fig. 7c). This finding was confirmed via histological evaluation (Fig. 7g,h) and also showed that the blood formed a large clot inside the pulmonary arteries (Fig. 7i). In marked contrast, revascularized lungs were able to circulate the blood into the peripheral region of the lung after orthotopic transplantation (Fig. 7e,). Blood was allowed to circulate for about 10 min after reperfusion. Lungs were then fixed for histological evaluation which showed blood perfusion in the most peripheral capillaries of the pulmonary vasculature (Fig. 7i,j). Red blood cells were observed in the alveolar area inside the lumens formed by CD31-positive HUVECs (Fig. 7j,k). Notably, we observed only a small alveolar hemorrhage in the recellularized peripheral lungs.

## Discussion

This study demonstrated for the first time that bioengineered lungs composed of human endothelial cells and decellularized murine lungs can be experimentally transplanted into adult mice. The decellularization efficiency of the cannulated lung scaffold was highly consistent and reproducible. Scaffold architecture and extracellular matrix components, such as collagen and laminin, were maintained after the decellularization procedure. Then, we developed a perfusion bioreactor system that is able to provide continuous media circulation using a pulsatile pump recapitulating the physiological blood flow ex vivo^17^. After the perfusion-based bioreactor culture, HUVECs formed vascular lumens from the proximal large-diameter pulmonary arteries to the peripheral capillary in the decellularized lung scaffold. Orthotopically transplanted mice that received scaffolds repopulated with 30 million HUVECs demonstrated homogeneous pulmonary blood perfusion in vivo.

With this mouse-scale bioengineering platform, we were able to conduct multiple experiments using significantly fewer cell resources compared to previous reports using larger animals^3–9^. In addition, smaller lungs made it easier to quantify the distribution of endothelial cells by whole slide scanning, TEM and quantitative image analysis. Quantitative gene expression analysis revealed relatively higher endothelial-related gene expression, such as CD31 and VECAD, after the dynamic perfusion culture compared to flat, static culture, suggesting the potential differentiation of HUVECs toward mature endothelial cells^18^. Although the difference could have been due to the expression change of the internal control, the potential phenotypic change of the endothelial cells should have been induced by the perfusion-based bioreactor culture. Taken together, this platform, however small, is biomimetic and relevant to allow adequate evaluation of revascularization protocols in vitro and in vivo*.*

One of the hurdles in vascular engineering is the optimal number of cells required to have sufficient coverage of the pulmonary vasculature of acellular lung grafts^19^. And for the most part, previous literature investigating the efficiency of vascular coverage in decellularized lungs has done so, using an arbitrarily chosen number of cells for injection^7,9,20^. The advantage of our system is that the tip of the vascular access catheter is directly inserted and secured in the main PA, which guarantees stable flow into the pulmonary circulation, unlike the previously reported method where a gavage needle was fixed in the right ventricle, which might not fully prevent the media leakage from the heart–lung block^14^. Actually, the numbers of cells that escaped from the scaffold at initial injection were comparable regardless of the number of cells injected. Using the more feasible mouse lung scaffold platform, we compared revascularization efficiency using lung scaffolds by injecting varying numbers of cells.

Lung fractal analysis has been previously used to quantify morphological changes in the presence of disease using clinically obtained computed tomography or histological images^16,21–23^. In this study, we used the box-counting method to better characterize the distribution and coverage of the endothelial cells in the scaffold^24^. The box-counting dimension for the macrostructures is smaller for the decell, 0.13–0.19, compared to 0.78 for the native mouse lungs. In the box-counting analysis of 2D images, a dimension closer to 2 indicates that the structures are distributed two-dimensionally, whereas a value closer to 0 suggests that the microstructure is sparsely distributed. Our analysis shows that in scaffolds repopulated with increasing numbers of cells, the box-counting dimension of HUVECs approaches those calculated for the native mouse lungs, indicating that the distribution of HUVEC microstructures approached the distribution of microstructures in the native mouse lungs. On the other hand, vascular coverage did not improve with cell numbers over 30 million cells, and endothelial cell coverage in the scaffold was highest when 30 million cells were injected (Fig. 5e).

Although the distal vascular area was not fully re-vascularized by HUVECs, TEM observation demonstrated the higher distribution of the endothelial cells toward the capillary vasculature (Fig. 6i). After the transplantation of the revascularized lung scaffold, we did not observe severe alveolar hemorrhage; a notable observation in previous studies^3,9^. We did however observe significant edema in the lung parenchyma. Given that endothelial cells injected from the PA appeared to cover only the pulmonary arteries and adjacent capillary vasculature, the severe edema was likely caused by the absence of pulmonary vein coverage^7^.

One of the limitations of this study is that the mouse-scale scaffold may not reproduce the microarchitecture of the human lungs, and that there may be minute changes in structure. Future experiments can include similar studies with human 3D lung slice cultures. Never the less, given that the overall architecture of the mammalian lungs is basically consistent across species regardless of the body size^25^, knowledge obtained in mouse-scale experiments can be adapted to human-scale lungs. On the other hand, the individual cell volume is almost identical across species^26^, suggesting that the narrower lumens formed by HUVECs in the capillary regions might be due to the difference between cultured cells and primary cells. Secondly, in this proof-of-concept study, we did not perform long-term perfusion culture of recellularized lungs. Cells can proliferate and transform in shape in the scaffold during the perfusion culture and the optimal cell numbers to be injected per lung can be different after long-term culture. Also, the viability and coverage of the cells should be confirmed in long-term perfusion culture.

In summary, despite these shortcomings of the current platform, the mouse-scale lung bioengineering platform developed in this study can answer the technical and biological questions allowing for quantitative analysis. This is especially true given its advantages with respect to cost and throughput in comparison to other larger systems such as rats, and swine. This platform can be easily extended to include re-epithelialization, where endothelial cells and epithelial cells will be physiologically positioned allowing investigation of the cell–cell interactions in future research.

## Materials and methods

### Animal husbandry

All animals in this study were maintained under a specific pathogen-free condition in the animal experiment facility at Tohoku University. All experiments were performed in accordance with the Regulations for Animal Experiments and Related Activities at Tohoku University (15^th^ edition), published by Tohoku University (https://www.clag.med.tohoku.ac.jp/clar/en/). This study was approved by the Institutional Animal Care and Use Committee at Tohoku University (#2020AcA-041-01). This study is reported in accordance with ARRIVE guidelines.

### Mouse surgery

C57Bl6 mice were purchased from Charles River (Japan). Male mice weighing between 25 and 30 g (12-week-old or older) were used for the surgery. Surgical procedures were performed under a surgical microscope M525 (Leica, Germany) or a stereomicroscope (Zeiss, Switzerland). Mice were euthanized with an overdose of isoflurane. The mice were fixed in the supine position. The abdominal and thoracic cavity were opened through the median incision. Then the inferior vena cava and the superior vena cava were ligated. The descending aorta was cut in the abdominal cavity to drain the blood, and then 3 mL of PBS was injected via the right ventricle to flush the pulmonary circulation. Once both lungs were adequately flushed, a 2 mm window was made under the tricuspid valve using scissors, and a 3F rat jugular vein catheter (#C30PU-RJV130, Instech/Primetech, Tokyo) was inserted into the pulmonary artery through the window. The catheter was fixed by ligating the root of the PA. Then an 18G I.V. catheter (Terumo, Tokyo) was inserted into the trachea and fixed by a ligation. Then the heart–lung block (HLB) was removed from the thoracic cavity after cutting down the adjacent vessels and tissues. The procedure was summarized in supplementary Fig. 1.

### Decellularization of the lung

Mouse lung decellularization was performed according to the previous literature with minor modifications^10^. Each liquid injection was performed through the PA or tracheal catheters. First, incubate the heart–lung blocks in distilled and deionized water (DDI) for 1 h at 4 °C. Then, 0.1% Triton-X (Sigma, Japan) in DDI was injected and incubated for 24 h at 4 °C followed by 24-h incubation with 2% sodium deoxycholate in DI. On the final day of the procedure, the heart–lung blocks were incubated with 1 M NaCl for 1 h at room temperature, followed by 1 h incubation with 0.1% DNaseI (Sigma, Japan). After rinsing with PBS, the heart–lung blocks were stored in PBS/antibiotics at 4 °C up to two weeks until use.

### Cell culture

Human umbilical vein endothelial cells (HUVEC, Lonza, Japan) were cultured using an EGM-2 Endothelial Cell Growth Medium-2 BulletKit (Lonza) as manufacturer’s instruction. Cells were maintained in a CO_2_ incubator at 37 °C. Cells between passage 2 and passage 5 were used for the experiments.

### Perfusion-based bioreactor assembly and perfusion organ culture

An in-house developed perfusion-based bioreactor was assembled as described in Fig. 3a. For gravity-driven cell injection, HUVECs were suspended in a glass bottle at a concentration of 1 × 10^6^/mL, and the bottle was placed approximately 30 cm above the organ chamber. A decellularized mouse heart–lung block was connected to the tubing through the PA catheter, and then, a stopcock was opened to allow the cells to be injected into the decellularized pulmonary vasculature. After the cell suspension was completely injected into the heart–lung block, the perfusion circuit was connected to a pulsatile pump (Masterflex L/S Digital Precision Modular Drive, Cole-Parmer, IL), and the media was perfused at a rate of 2 mL/min (or 6 rpm) using a Benchtop Controller (Cole-Parmer).

At the end of the perfusion-bioreactor culture, the recellularized heart–lung block was divided into individual lobes, and the lobes were either fixed in 10% neutralized formalin or immersed in RNAlater (ThermoFischer Scientific) and stored in a -80 °C freezer.

### RNA extraction and qPCR

The frozen tissue was later homogenized in Buffer RLT (Qiagen, Japan) using TissueLyserII (Qiagen). Total RNA was extracted using an RNeasy mini kit (Qiagen) according to the manufacturer’s instructions. cDNA was reverse transcribed using iScript reverse transcription supermix (Biorad Laboratories, Japan) according to the manufacturer's instructions. qPCR was performed using ssoAdvanced Universal SYBR Green Supermix (Biorad Laboratories) with CFX96 Real-Time PCR Detection System (Biorad Laboratories). The primers used for this study are; PrimePCR™ SYBR® Green Assay GAPDH, Human (Unique Assay ID: qHsaCED0038674), CD34, Human (Unique Assay ID: qHsaCID0007456), CDH5, Human (Unique Assay ID: qHsaCID0016288), KDR, Human (Unique Assay ID: qHsaCID0006310), and Pecam1 (Forward sequence: CCACGCCTAGCCAAAATCAC, Reverse sequence: CATGTGGCCCCTCAGAAGAC), all purchased from Biorad Laboratories.

### Histology

Formalin-fixed and paraffin-embedded tissue was cut into 3 µm slices. Hematoxylin–eosin staining (HE), Masson’s trichrome staining (MT), and combined Verhoeff and Masson trichrome staining (VMT) were performed under the standard protocols. For immunohistochemistry, a heat-induced antigen retrieval procedure was performed using an Histofine antigen-retrieval solution pH 9 (Nichirei Bioscience, Japan) by incubating sections under 120 °C degree for 15 min in an autoclave. Sections are then incubated with a specie-matched blocking serum for 30 min, followed by an overnight incubation with a primary antibody. The following day, antibody reactions are visualized using Histofine Simple Stain MAX PO (Nichirei Bioscience) and DAB chromogen (Nichirei Bioscience). For immunofluorescence of CD31, an AlexaFluore 488 goat anti-rabbit IgG was applied to the tissue section after the overnight incubation of a primary andibody for CD 31. The sections were then mounted using ProLong Gold antifade mounting medium with DAPI (ThermoScientific, MA). Primary antibodies used in this study are; rabbit anti-human CD31 polyclonal antibody (1:50, Santa Cruz, TX), mouse anti-human alpha-smooth muscle actin antibody(1:50, BioLegend, CA), rabbit anti-human laminin polyclonal antibody (1:300, Abcam, UK).

Images were digitally captured using SLIDEVIEW VS200 (Olympus/Evident, Japan) with a 200 times magnification. Images were processed using the OlyVIA image processing software (Olympus/Evident). For immunofluorescence imaging, fluorescent signals were captured using Leica confocal lase microscope (TSC Sp8, Leica) and Las X image processing software (Leica).

### Image analysis

The pixel number of the scaffold area was counted by selecting the edge of the scaffold (Fig. 5b). Then, the HE images of decellularized, recellularized, and native lungs (Fig. 5a and Supplementary Fig. 2) were converted into binarized images and made pixels transparent according to the brightness threshold above 190 and the gray scale value as 35 (Fig. 5c). The number of black pixels of the converted images was counted, and the percentage of pixel counts of the cells to the scaffold area was used as a vascular coverage of HUVECs (Fig. 5d). Then, the box-counting (Fig. 5e) was performed using the image analysis program PoreSpy (https://porespy.org/)^27^ using the converted images. The results were plotted in log–log graphs to estimate fractal dimensions, and the fractal dimension of each image was calculated as a slope of the linear fit line of these plots (Fig. 5f and Supplementary Fig. 2).

The difference in cell coverage was tested using one-way ANOVA. And the fractal dimensions of decellularized and recellularized mouse lungs were analyzed with Kruskal–Wallis test followed by Dunn’s multiple comparison test using Prism (Version 10, GraphPad).

### Transmission electron microscopy

Decellularized and recellularized lung tissues were perfused with a fixative composed of 2% of paraformaldehyde, 2.5% of glutaraldehyde, and 0.1 M cacodylate buffer overnight at 4 °C. The tissues were cut into small pieces and incubated with 0.1 M cacodylate buffer for 24 h at 4 °C. Then the tissues were further fixed with 1% osmium tetroxide for 90 min and dehydrated in a graded series of ethanol. For micro-sectioning, the tissues were embedded into epoxy resin and cut into 80 nm slices using Leica EM UC-7. Finally, the sections were stained with 2% uranium acetate, followed by a 1% mixture of lead acetate, lead nitrate, and lead citrate. Images were captured using an electron microscope JEM-1400 (Japan Electron Optics Laboratory, Japan). To quantify the distribution of HUVECs in different vascular locations, total of 169 cell positions were captured. The caliber of the capillary vessel to which an individual endothelial cell was attached was represented as the diameter of the circle inscribing the vascular scaffold (Fig. 6h and Supplementary Fig. 3, orange dotted circles).

#### Orthotopic transplantation of the bioengineered lung

Orthotopic bioengineered mouse left lung transplantation was performed using our previously described cuff technique^28,29^, with modifications. Briefly, after a three-day perfusion bioreactor culture, the left pulmonary hilum of the bioengineered lung was dissected. A cuff made with a 24G angiocatheter was attached to left pulmonary artery, and cuffs made with a 20G angiocatheter were attached to the pulmonary vein and bronchus. Recipient mice were anesthetized with an intraperitoneal injection of a triple mixture of medetomidine hydrochloride (0.75 mg/kg), midazolam (4 mg/kg) and butorphanol tartrate (5 mg/kg) and were intubated orally with a 20G angiocatheter and maintained under mechanical ventilation (Harvard 687, Harvard Apparatus) with 0.5 mL of tidal volume, 120/ min frequency, 2 cmH_2_O of positive end-expiratory pressure, and 100% of inhaled oxygen concentration. A left thoracotomy is performed through the third intercostal space. After dissection of left hilum, a slip knot with 9–0 silked was used for pulmonary artery and a microvascular clamp was placed to the bronchus together with the pulmonary vein. Then left lung transplantation was performed, and the bioengineered lung was re-perfused. We performed two transplantation surgeries using bioengineered left lungs. A decellularized lung scaffold was used for cell-free control (n = 1). After reperfusion, blood was allowed to circulate for 30 min. Then, animals were sacrificed, and the graft was harvested for histological observation.

### Supplementary Information

Supplementary Figures.

## Data Availability

The datasets generated during the current study are available from the corresponding author on reasonable request.
