# Supplementary material for: Orthotopic transplantation of the bioengineered lung using a mouse-scale perfusion-based bioreactor and human primary endothelial cells
Source: Sci Rep. 2024 Apr 4;14:7040. doi: 10.1038/s41598-024-57084-0 (PMC10994903; doi:10.1038/s41598-024-57084-0)
Supplement: Supplementary file 1 — Supplementary Figures. [file 41598_2024_57084_MOESM1_ESM.pdf]

Supplementary Figure 1

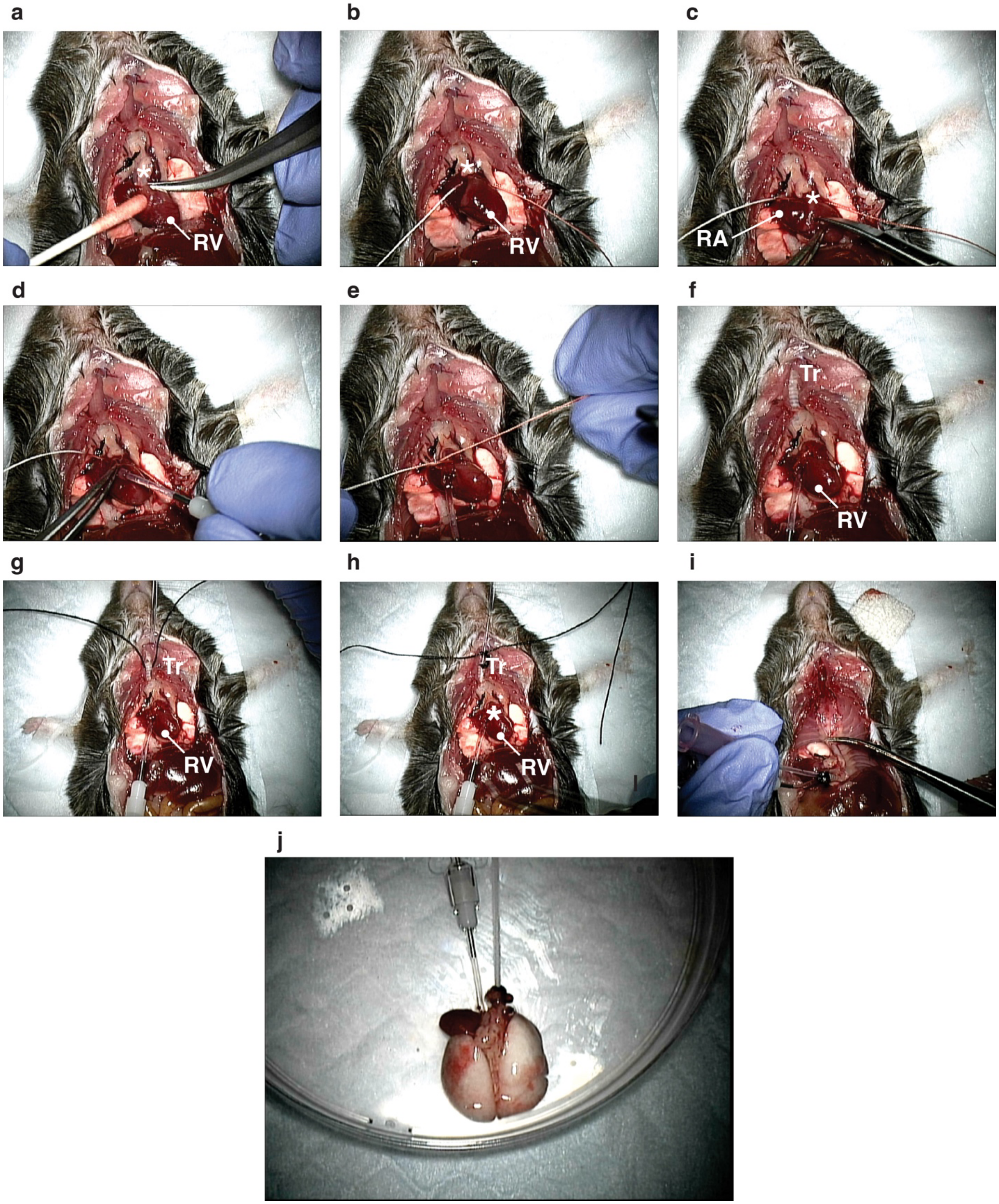

Supplementary Figure 1. Mouse surgery

The thoracic cavity of the mouse was opened, and the pulmonary artery was ligated by a silk suture (a and b). The access window for the pulmonary artery (PA) was cut on the right ventricle wall immediately below the pulmonary artery (c). The PA catheter was inserted (d). The PA catheter was fixed by ligating the silk suture (e). The trachea was exposed (f). The trachea was taped by a silk suture, and the tracheostomy was performed (g). The tracheal catheter was inserted and fixed (h). The heart-lung block was removed from the thoracic cavity (i). The isolated heart-lung block was transferred to the next procedure, such as decellularization (j). * Main pulmonary artery, RV: Right Ventricle, RA: Right Atrium, Tr: Trachea.

Supplementary Figure 2

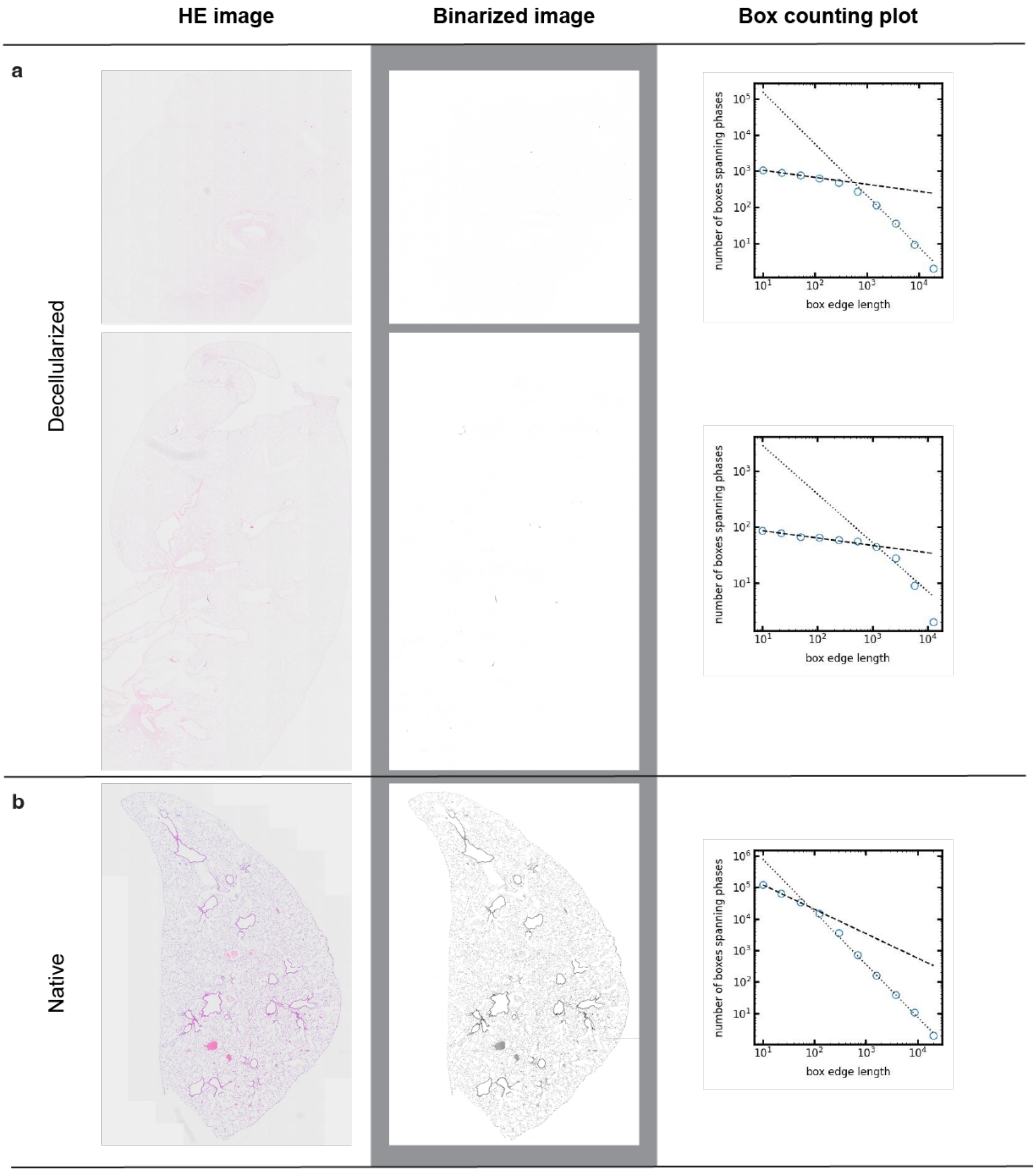

(continued)

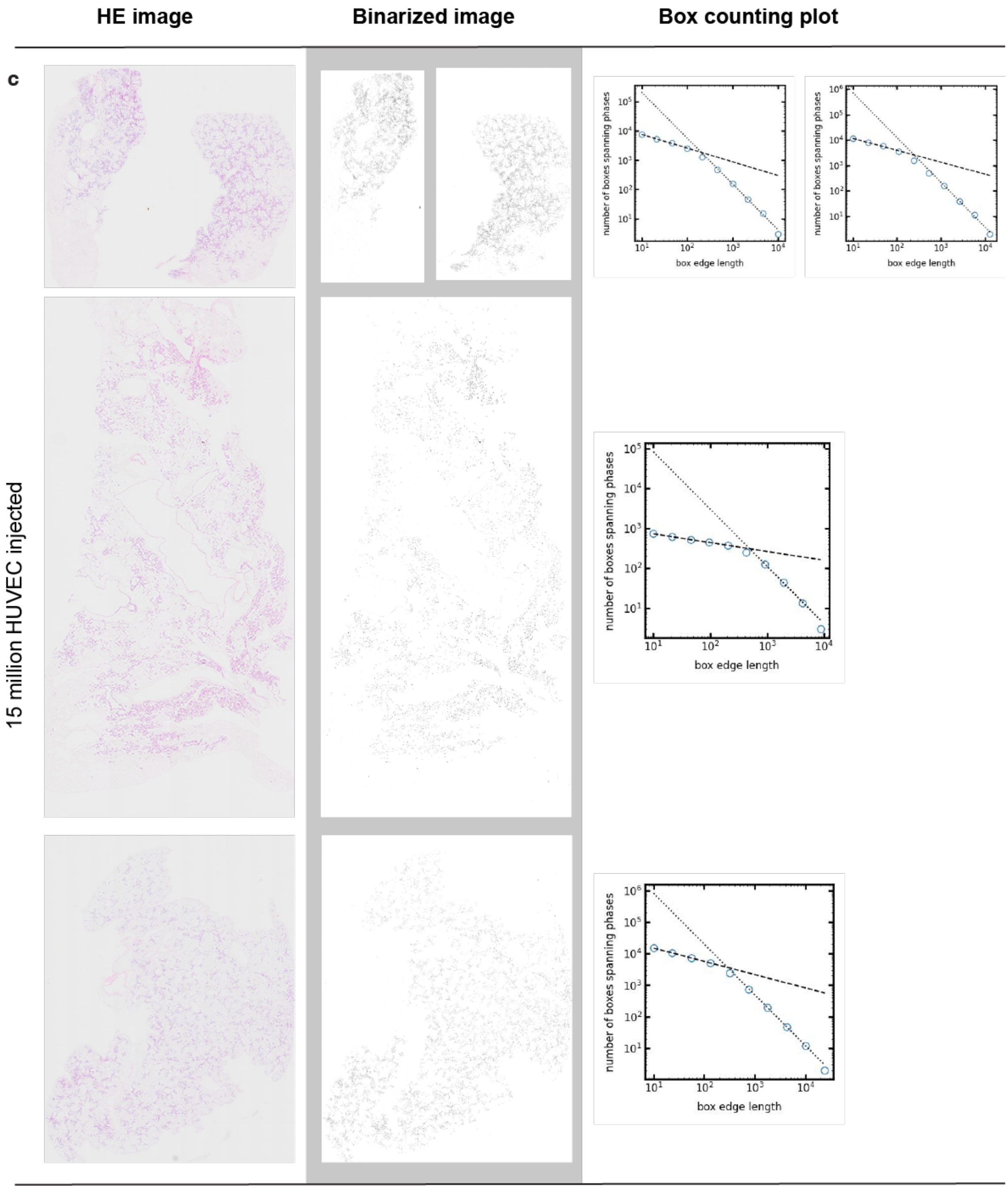
HE image
Binarized image
Box counting plot
c
15 million HUVEC injected
number of boxes spanning phases
box edge length

(continued)

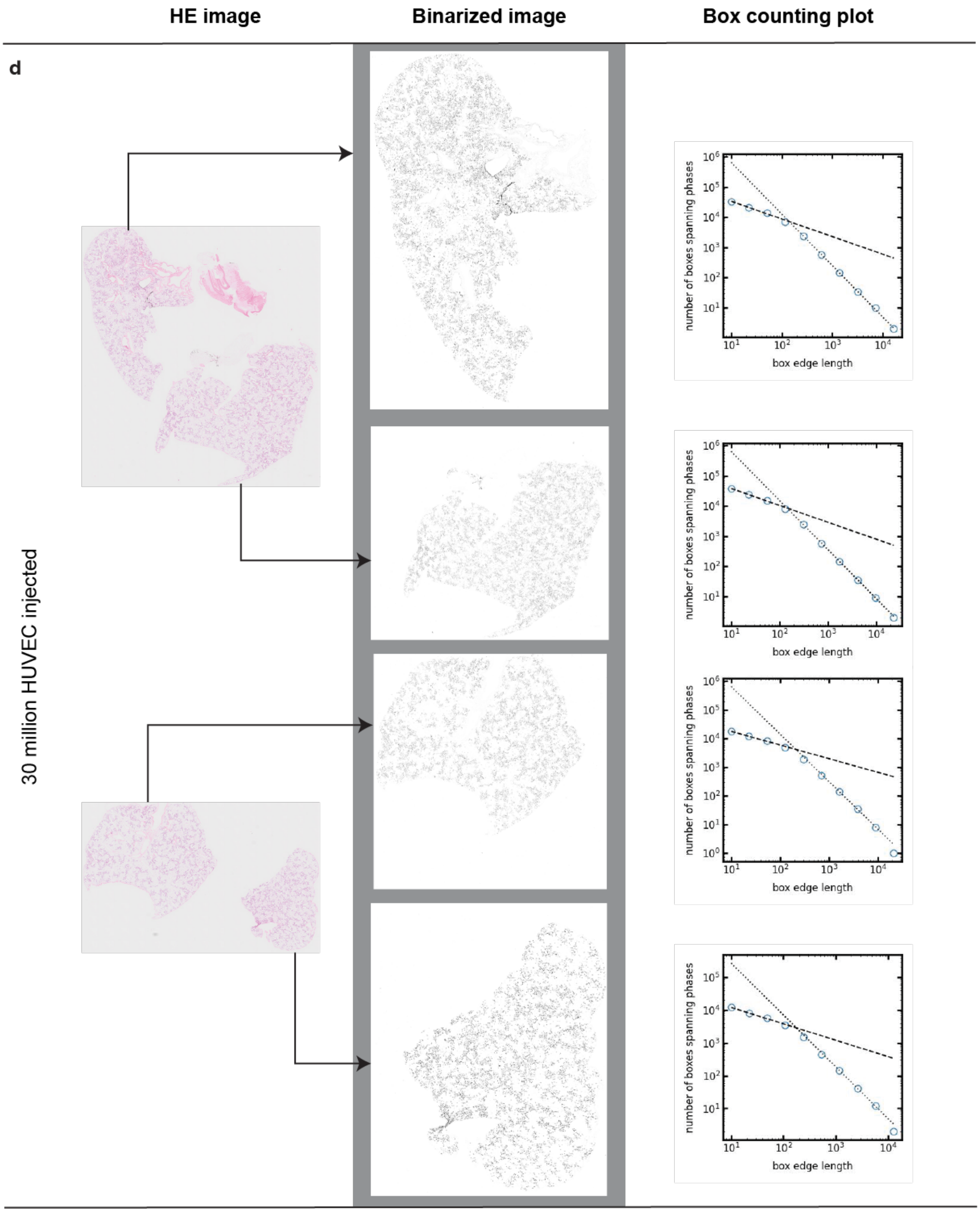

HE image
Binarized image
Box counting plot
d
30 million HUVEC injected
number of boxes spanning phases
box edge length

(continued)

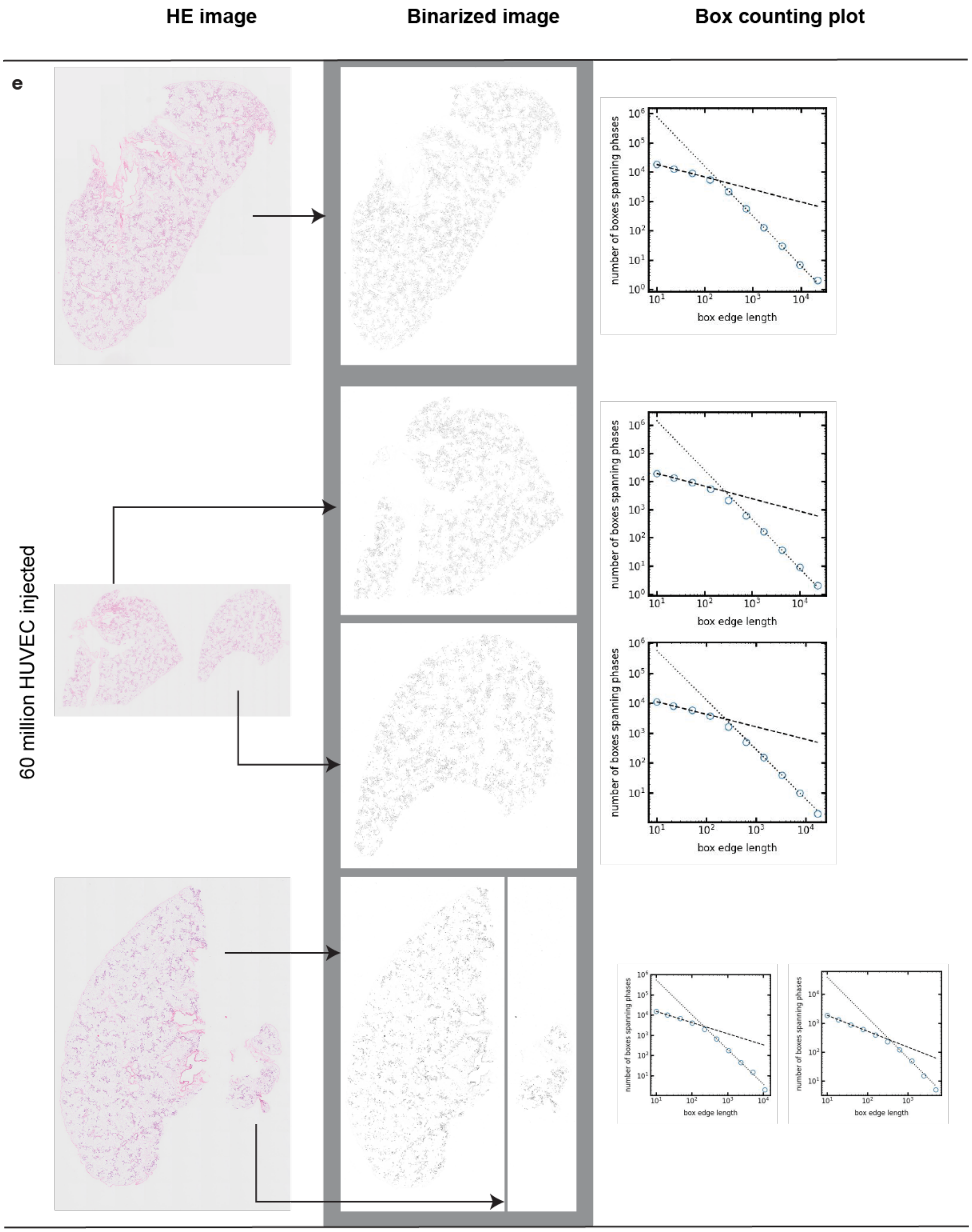

Supplementary figure 2 Box counting of native, decellularized, and recellularized lungs

The left column represents the H and E staining of the samples. The middle column represents the binarized image of the corresponding HE images. The right column represents the log-log plots using the box-counting methods.

Supplementary Figure 3

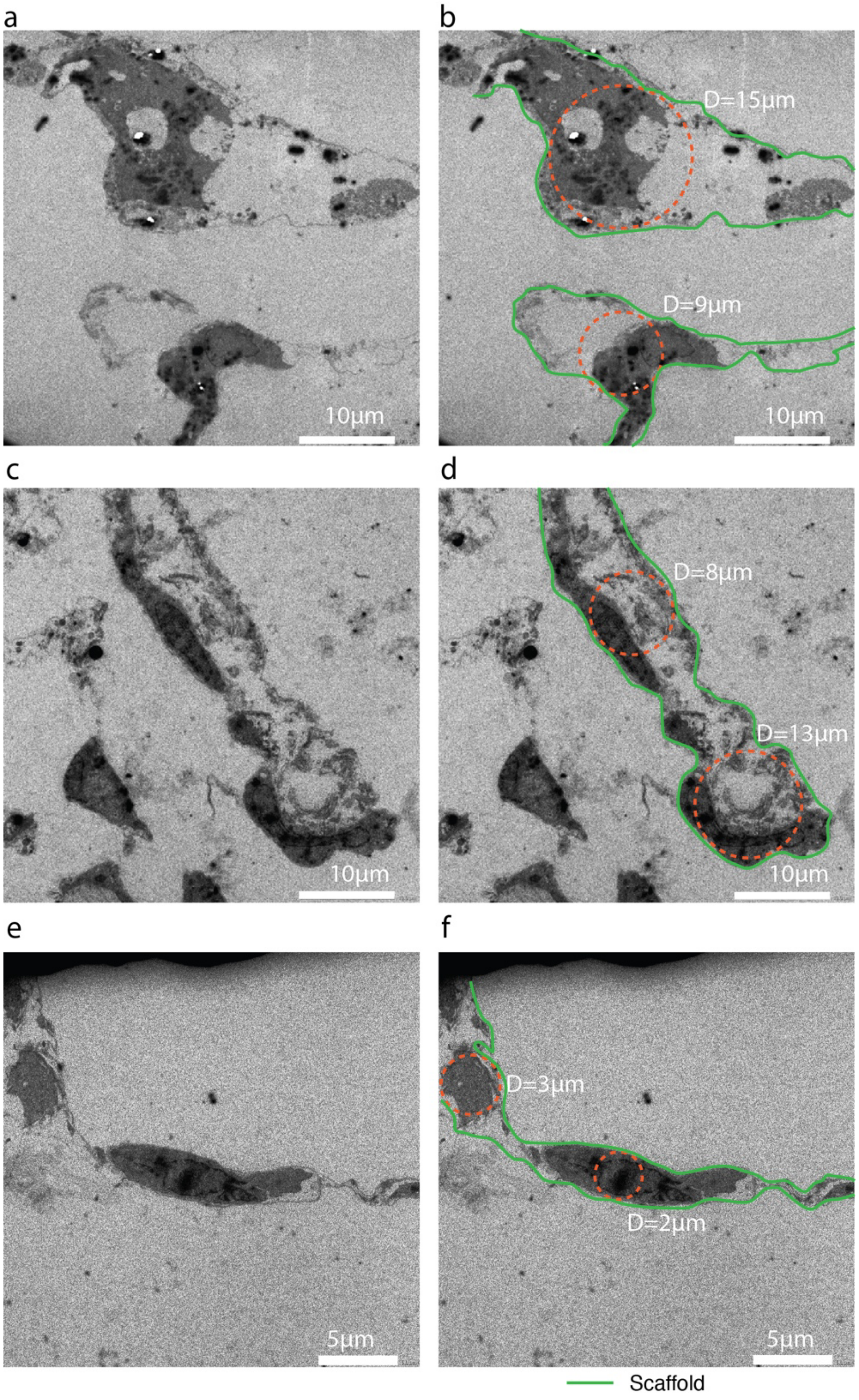

Supplementary Figure 3 Measurement of the vascular diameter

Representative images of transmission electron microscope of the engineered lungs (a, c, and e). Each dotted circle represents the cariber of the vascular tube (indicated by green lines) where an individual endothelial cell was attached (b, d, and f). D: Diameter.
